# Factors associated with major complications defined by Clavien–Dindo classification 3–5 after liver transplantation: a prospective multicenter cohort study

**DOI:** 10.3389/fsurg.2025.1648512

**Published:** 2025-11-14

**Authors:** Antoni Sabate, Marta Caballero, Rosa Gutierrez, Lourdes Pérez, Julia Vidal, Sandra Llaurado, Pilar Hereu, Judith Peñafiel, Annabel Blasi

**Affiliations:** 1 Department of Anesthesiology, University Hospital of Bellvitge, University of Barcelona Health Campus, IDIBELL, Barcelona, Spain; 2 Department of Anesthesiology, University Hospital of Cruces, Bilbao, Spain; 3 Department of Anesthesiology, Clinic Hospital, University of Barcelona Health Campus, IDIBAPS, Barcelona, Spain; 4 UICEC, UBIDI, University of Barcelona Health Campus, IDIBELL, Barcelona, Spain

**Keywords:** acute kidney injury, Clavien–Dindo postoperative complications, liver transplantation, morbidity, mortality

## Abstract

**Background:**

We aimed to explore factors preoperative and intraoperative, associated with Clavien–Dindo classification 3–5 (CDC 3–5) after LT.

**Methods:**

Secondary analysis of multicenter prospective cohort data for 305 consecutive patients. European Clinical Trials Database -EudraCT 2018-002510-13. The primary outcome was the incidence of CDC 3–5 complications recorded during the ICU stay. We used a log-binomial regression model to evaluate associations.

**Results:**

Cardiac-death donors provided 30.16% of grafts. Vena cava preservation was performed in nearly all patients, and a temporary portacaval shunt was used in 41.91%. Intraoperative red blood cell (RBC) transfusion was required in 51.48%, and 27.37% required additional transfusions within 24 h of LT. CDC 3–5 status occurred in 97 patients (31.80%, 95% CI, 26.58%–37.03%). Thrombotic events occurred in 19 patients (6.25%): 6 in portal veins, 5 in hepatic arteries, 2 in mesenteric veins, and 6 in deep veins. Two patients were retransplanted. Twenty-two (7.21%) required reinterventions; 2 were retransplanted; and 20 (6.57%) were readmitted to the ICU. Adjusted relative risk (aRR) calculation found associations with a MELD score >23 (aRR, 1.92; 95% CI, 1.28–2.8), baseline hemoglobin concentration (aRR, 0.98; 95% CI, 0.97–0.99), zero RBC transfusion (aRR, 0.37; 95% CI, 0.28–0.72), an RBC transfusion cut point of >2.5 (aRR, 1.96; 95% CI, 1.29–2.96), PRS (aRR, 2.11; 95% CI, 1.43–3.10), and fibrinogen administration (aRR, 1.07; 95% CI, 1.05–1.09). We found no associations with temporary portocaval shunt (aRR, 1.02; 95% CI (0.7–1.48), cold and warm ischemia times or surgical time and intraoperative fluid administration.

**Conclusion:**

We conclude that PRS at reperfusion of the liver graft and the volume of RBCs transfused are the main modifiable factors that influence major complications reflected by CDC 3–5 status after LT.

**Clinical Trial Registration:**

https://clinicaltrials.gov/, identifier NCT04405518.

## Background

Liver transplantation continues to be associated with high risk for postoperative complications (1–3). Organ shortages have prompted greater utilization of higher risk grafts, in parallel normothermic and hypothermic oxygenation perfusion machines are today more common from cardiac-donor procurements (4, 5).

Scores as donor risk index (6, 7) have been published and models combining MELD score (Model for End-Stage Liver Disease) have been reported to predict complications, with lower mortality in recipients with high MELD scores (8–10).

We hypothesized that beyond the short- and long-term influence of the risk factors earlier studies have identified, certain other modifiable factors may be relevant although they have not been extensively analyzed in prospective series set in the context of today's advances in surgical and donor procurement processes. We aimed to explore all modifiable risk factors preoperative and intraoperative associated with major complications after LT in a series of consecutive recipients registered prospectively in three centers.

## Methods

Data from all consecutive adults aged 18–80 years who were scheduled for LT in three centers were assessed from August 2, 2019, to November 2, 2021. The only criterion for excluding a patient was cancellation of the procedure. The extended protocol was approved by the institutional review board (IRB) of the lead hospital (University Hospital of Bellvitge, approval number AC 033/18). Human Ethics and Consent to Participate declarations are validated by the IRB. It was registered in the European Registry in 2018 (European Clinical Trials Database -EudraCT 2018-002510-13) and in ClinicalTrials.gov (NCT04405518, first registration date, 10-10.2019). Consent to participate: Every human participant has provided their consent. Methods were performed in accordance with the relevant guidelines and regulations. The work has been reported in line with the STROCSS guidelines (11).

### Graft and anesthesia management, surgery, and transfusion protocols

The details of the management protocols have been published in a previous controlled trial (12). Briefly, organ procurement from controlled cardiac-death donors were made by normothermic regional perfusion (13). Vena cava preservation was attempted in all patients. If exceptionally such preservation was not feasible, a venovenous bypass or a complete caval clamp was used. At the dissection stage, a temporary portocaval shunt (PCS) was performed in patients without total portal thrombosis and/or spontaneous portal derivation (14). In this procedure, the portal vein was cross-clamped and divided; then, after exposure and lateral clamping of the infrahepatic vena cava, the proximal end of the portal vein was anastomosed end to side with the vena cava by using a running suture. The PCS was maintained during the anhepatic stage and was taken down prior to suprahepatic anastomosis.

Hemostatic management was guided by thromboelastometry. Infusion criteria were as follows: RBCs to maintain hemoglobin above 80 g/L, platelet concentrates if a count fell below 30,000/mm^3^, and intravenous tranexamic acid boluses of 500 mg if fibrinolysis (>15% lysis at 60 min) was detected by thromboelastometry for fibrin function (FibTem). Cell saver devices were not used.

At the end of surgery, all patients remained mechanically ventilated on transfer to a postoperative intensive care unit (ICU). Tracheal extubation criteria were explained in a previous article (15).

All patients were administered a fourfold immunosuppression regimen. Specifically, a methylprednisolone bolus of 500 mg was given at the time of reperfusion; thereafter, low-dose doses of prednisone were individually tapered. Mycophenolate, basiliximab, and tacrolimus were adjusted to clinical needs and monitored by measuring whole blood levels.

### Primary outcome, other outcomes of interest, and risk factors

The primary outcome was the incidence of a composite of major postoperative complications defined by a Clavien–Dindo classification (16) of 3–5 (CDC 3–5). The secondary outcomes were the incidence of acute kidney injury (AKI) classified as grade 2 or 3 according to the guidelines of the Kidney Disease: Improving Global Outcomes organization (17), the incidence of intra- and postoperative thrombotic events in the graft or legs (assessed by Doppler ultrasound), and in the lung (assessed by computed tomography). Retransplantations and mortality within 90 days were recorded in the patient's electronic case record form, along with all relevant data and adverse events. The data monitoring committee also reviewed all adverse events, and an annual safety report was sent to the Spanish Agency for Medicines and Medical Products and all IRBs that approved the protocol.

Variables considered as possible risk factors included recipient and donor characteristics, intraoperative data related to LT, and anesthetic management. Recipient characteristics were age; sex; body mass index; diabetes mellitus; hypertension; cardiac disease; respiratory disease; indication for LT; MELD score; Child score; UNOS status; hemoglobin, creatinine, plasma fibrinogen levels; and the international normalized ratio of prothrombin time (PT/INR); platelet count; and baseline thromboelastometry profile. Donor characteristics were type of donor (after brain or cardiac death), donor age, and cold ischemia time (CIT). Intraoperative data were surgical time; warm ischemia time (WIT); the use of PCS, infusions of blood components, fibrinogen concentrate, tranexamic acid, crystalloids, and albumin; and the development of postreperfusion syndrome (PRS).

### Statistical analysis

Descriptive statistics for patients and surgeries were expressed as mean (SD) for discrete variables and median [interquartile range (IQR) or range] for continuous variables. Categorical variables were expressed as number of cases and percentages. Statistics related to actuarial graft and patient survival were also compiled. For analysis of the study's primary and secondary outcomes, the cohort was stratified according to CDC status (0–2 vs. 3–5). Parametric or nonparametric tests were used for continuous variables according to normality or non-normality of distribution. For categorical variables, chi-square tests or Fisher exact tests were used.

We used a log-binomial regression model to evaluate the associations between the potential risk factors and CDC 3–5 status. Complications were defined as events occurring during the ICU stay. Given this definition, events occurring after discharge from the ICU that led to readmission to the ICU, were not included in the analysis to simplify the model and avoid the need for right-censoring or time-to-event analysis. To address the potential issue of collinearity, we calculated the variance inflation factor for all variables. Any variables found to be collinear were excluded from the regression analysis. We analyzed RBC transfusion both as a continuous variable and a dichotomic one (0–6 units vs. massive transfusion, i.e., >6 units) to find the best cut point related to the primary outcome. Risk was adjusted for MELD score based on its possible positive associations with the dependent outcome variables. Relative risk (RR) and adjusted RR (aRR) and 95% CIs were also calculated. All analyses were performed with the statistical software package R, version 4.1.0 for Windows (http://www.R-project.org, The R Foundation).

## Results

A total of 318 patients on the LT waiting list were initially evaluated; seven patients were removed from the list, and six procedures were cancelled in the operating room. Finally, data for 305 patients were analyzed. Table 1 shows patient and surgical data for the cohort, stratified according to CDC status. The median age was 60 years (IQR, 55–64 years), 77.38% were male, and the most common diagnosis was alcohol cirrhosis 49.51%, followed by hepatocarcinoma (22.30%). The median MELD score was 17 (IQR, 11–21), 9.84% had partial portal thrombosis, and the median hemoglobin value was 108 g/L (IQR, 88–126). A prior history of abdominal surgery was present in 32.39% of patients.

**Table 1:** Patient characteristics and surgical data.

Variables	*N* = 305	CDC grade, 3–5 (*n* = 97, 31.8%)	CDC grade, 0–2 (*n* = 208, 68.2%)	*P* value
Patient characteristics				
Age (years)^b^	60.0 (55.0–64.0)	60.0 (55.0–66.0)	59.0 (54.0–64.0)	0.256
Male/Female	77.38%/23.62%	76.29%/23.71%	77.88%/22.12%	0.870
BMI (kg·m^2^)^a^	27.27 (4.90)	276.95 (5.65)	27.39 (4.66)	0.762
Diagnoses, preoperative data				
Indications for LT				
Alcoholic cirrhosis	49.51%	51.55%	48.56%	0.716
NASH	9.84%	11.34%	9.13%	0.692
Hepatocarcinoma	22.30%	15.46%	25.48%	0.070
Others	18.35%	21.65%	16.83%	0.454
Prior abdominal surgery	32.39%	40.30%	27.52%	0.111
Diabetes	32.95%	32.84%	33.03	1.000
Abnormal echocardiogram	16.48%	16.42%	16.51%	1.000
Pulmonary disease	17.61%	19.40%	16.51%	0.776
Ascites + pleural effusion	40.98%	53.60%	35.09%	0.049
Sodium (mEq/L)^b^	137 (133–140)	136 (130–139)	138 (133–140)	0.070
Preoperative kidney dysfunction	26.4%	29.85%	23.85%	0.482
Glomerular filtration rate (mL/min)^a^	93.36 (39.7)	88.72 (40.36)	96.21 (39.20	0.229
Creatinine (mg/dl)^b^	0.91 (0.74–1.15)	0.95 (0.77–1.23)	0.88 (0.74–1.11)	0.068
MELD score^b^	17 (11–21)	19 (11–24)	16 (10–21)	0.005
Child–Pugh score				0.017
A	30.74%	20.21%	35.64%	
B	30.74%	31.91%	30.20%	
C	38.51%	42.87%	34.16%	
UNOS classification				0.134
At home	60.33%	56.70%	62.02%	
On ward	30.10%	28.87%	30.77%	
ICU	9.51%	14.43%	7.21%	
Hemoglobin (g/L)^b^	108.0 (88–126)	92.0 (82–111)	115.0 (93.5–130)	0.001
PT/INR^b^	1.41 (1.24–1.71)	1.58 (1.31–2.01)	1.38 (1.19–1.66)	0.001
Platelet count (10^3^/mm^3^)^b^	79.0 (53.0–122.50)	76.0 (52.5–113.00)	83.0 (53.8–124.0)	0.001
Fibrinogen (g/L)^b^	2.30 (1.50–3.20)	2.00 (1.41–2.79)	2.52 (1.60–3.34)	0.001
ExTem^b^				
Coagulation time (s)	65.0 (60–75.0)	67.0 (60–77.0)	65.0 (60–75.4)	0.390
MCF (mm)	53.0 (46.0–62.0)	53.0 (44.0–60.25)	54.0 (47.0–62.0)	0.215
Lysis (%)	0 (0–0)	0 (0–0)	0 (0–0)	0.807
A10 FibTem MCF (mm)	11 (7.0–15.0)	11 (7.0–15.25	11 (8.0–15.0)	0.449
Donor type				
Brain death	69.84%	70.10%	69.71%	0.830
Cardiac death	30.16%	29.90%	30.29%	
Donor age (years)^c^	59.49 (18–84)	59 (25–84)	59 (18–78)	0.855
Preservation cava vein	95.71%	94.85%	96.15%	0.560
Venous bypass and cava clamp	4.29%	5.15%	3.85%	0.335
Temporary portocaval shunt	41.91%	43.30%	41.35%	0.844
Length of surgery (min)^b^	406 (320–1,443)	420 (330–1,436)	396 (310–1,451)	0.416
Cold ischemia time (min)^b^	377 (293–445)	384 (320.4–484)	370 (281–441)	0.114
Warm ischemia time (min)^b^	40.00 (30.00–54.00)	39.50 (30.00–56.00)	40.00 (30.00–52.00)	0.784
Reperfusion syndrome	38.49%	56.25%	30.29%	0.001
Transfusion during LT				
RBC (units)^b^	1 (0–3)	2 (0–5)	0 (0–3)	0.001
RBC risk cut point > 2.5 units	32.13%	49.48%	24.04%	0.001
Patient RBC required, by units				0.001
0	48.52%	29.90%	57.21%	
1–6	44.26%	57.73%	37.98%	
>6	7.22%	12.37%	4.81%	0.032
Fresh frozen plasma	10.16%	17.53%	6.73%	0.007
Apheresis platelets	12.46%	20.62%	8.65%	0.006
Fibrinogen concentrate (g)	2.00 (0.00–5.00)	4.00 (0.00–9.00)	0.00 (0.00–4.00)	0.001
Tranexamic acid, Yes	43.23%	49.48%	40.38%	0.171
Crystalloids + albumin (ml)^b^	2,250 (1,512–4,000)	2,281 (1,512–4,287)	2,100 (1,512–3,812)	0.452
Total transfusion during LT + 24 h after				
RBCs (units)^b^	1 (0–4)	4 (1–7)	0 (0–3)	0.001
RBCs required, by units				0.001
0	42.95%	21.65%	42.95%	
1–6	43.28%	50.52%	39.90%	
>6	13.77%	27.84%	7.21%	

ExTem, extrinsic thromboelastometry for fibrin tissue factor activation; FibTem, thromboelastometry for fibrin tissue factor activation and platelet inhibition; ICU, intensive care unit; MCF, maxim clot firmness; MELD, model for end-stage liver disease; NASH, nonalcoholic steatohepatitis; PT, prothrombin time; PT/INR, international normalized ratio of PT; RBC, red blood cells; UNOS, United Network for Organ Sharing.

Data are percentages of patients, unless otherwise indicated as mean (SD)^a^, or median (interquartile range)^b^, or median (range)^c^.

Donor age ranged from 18 to 84 years, and 30.16% of the patient's received grafts from controlled cardiac-death donors. Vena cava preservation was performed in nearly all patients, and a temporary PCS was used in 41.91% of the patients. Intraoperative RBC transfusion was required by 51.48%, and 27.37% required additional transfusion in the first 24 h after LT. During LT, 54.43% required infusion of fibrinogen concentrate. At reperfusion stage, 27.87% received tranexamic acid because of bleeding. Reperfusion syndrome was present in 38.49%.

The primary composite outcome of major postoperative complications indicated by CDC 3–5 status occurred in 97 patients (31.80%, 95% CI, 26.58%–37.03%). Table 2 shows surgical events and outcome of patients. Miscellaneous complications (pneumothorax, cardiac arrythmia, neurotoxicity) were registered in 12 patients. One patient died during the surgical procedure, five patients died within 30 days, one patient died on day 60, and another patient died on day 75. Two patients were retransplanted because of primary graft failure; both were discharged alive.

**Table 2:** Surgical events, and outcomes.

All patients	*N* = 305
Clavien–Dindo 3–5 (*n*)	97 (31.80%, 95 CI: 26.58%–37.03%).
Acute renal failure (*n*)	53 (17.43%, 95 CI: 13.30%–22.11%)
Thrombotic complications (*n*)	19 (6.25%)
Portal vein	6
Hepatic artery	5
Mesenteric vein	2
Deep vein	6
Infective non pulmonary complications	31 (10.16%)
Pulmonary complications	36 (11.9%)
Reoperations (*n*)	22 (7.21%)
Postoperative bleeding	15
Abdominal abscess	3
Biliary cause	4
Re-Transplantation (*n*)	2 (0.65%)
Death (*n*)	8 (2.62%)

**Figure 1:**
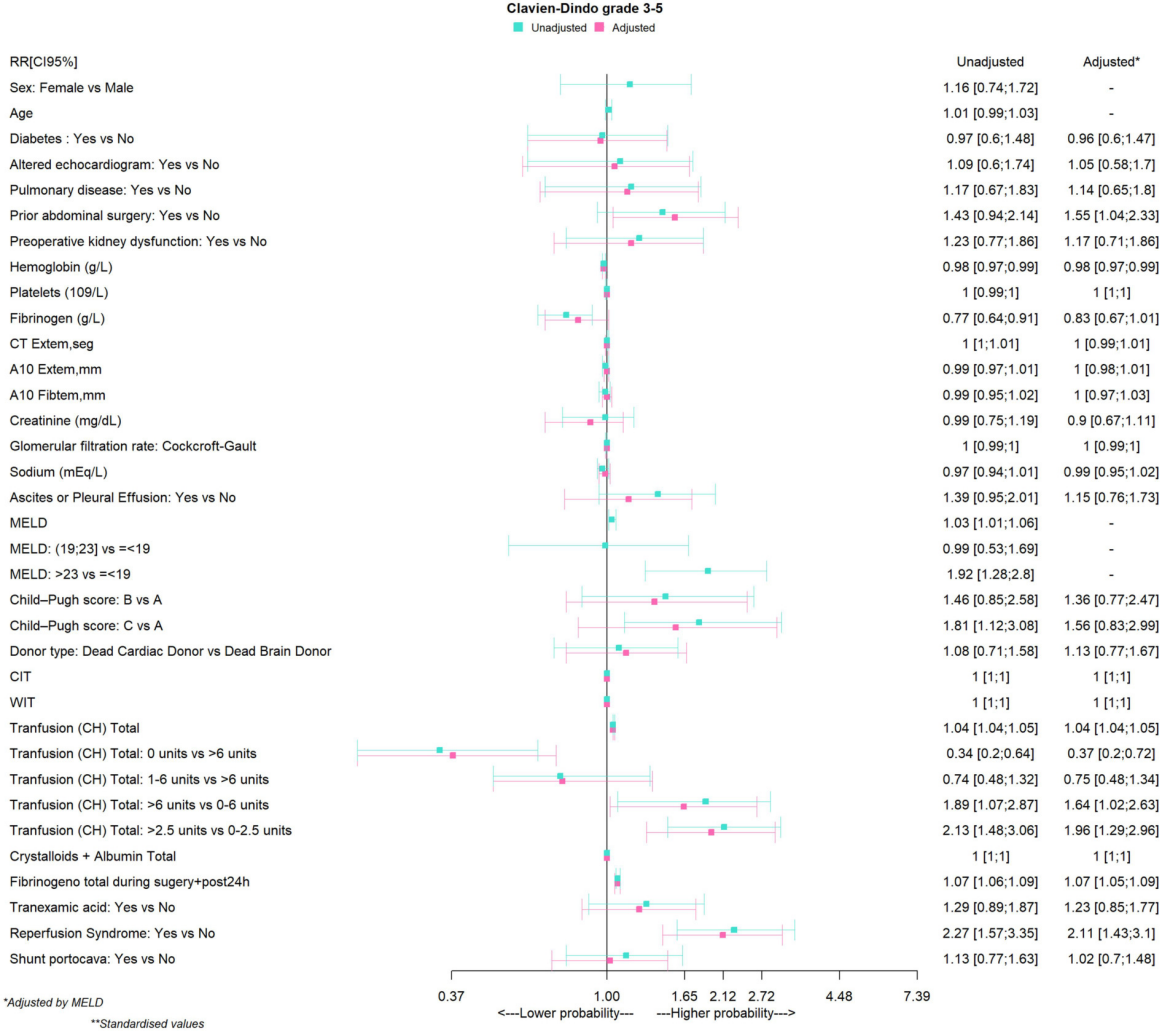
Relative risk (RR and aRR) conferred by factors associated with major complications after liver transplantation. A10 refers to amplitude at 10 min in ExTem or FibTem; CIT, cold ischemia time; CT, coagulation time; DBD, brain death donor; DCD, cardiac death donor; ExTem, extrinsic thromboelastometry for fibrin tissue factor activation; FibTem, thromboelastometry for fibrin tissue factor activation and platelet inhibition; MELD, model for end-stage liver disease; WIT, warm ischemia time.

The ICU length of stay was less than 1 week in 87.90%, between one and 2 weeks in 7.74%, and more than 2 weeks in 4.63%. Twenty patients (6.57%) were readmitted to the ICU. These patients were excluded from the binomial analysis, which was performed on data for 285 patients. The PT/INR ratio showed a variance inflation factor of 6.26 with the MELD score and was therefore excluded from the log-binomial regression analysis. Data used for the binomial regression model is presented in Table 3. The RR and aRR values derived from the binomial regression model for CDC 3–5 status are shown in Figure 1.

**Table 3:** Data used for the binomial regression model.

Variables	CDC grade, 3–5 (*n* = 80)	CDC grade, 0–2 (*n* = 205)	RR, (95% CI)	aRR (95% CI)
Patient characteristics				
Age (years)^a^	59.5 (55.0–66.0)	59.0 (54.0–64.0)	1.01 (0.99–1.03)	1.01 (0.99–1.03)
Male/Female	73.75%/26.25%	77.5%/22.5%	1.16 (0.74–1.72)	1.1 (0.73–1.66)
Preoperative data				
Prior abdominal surgery	40.67%	27.77%	1.43 (0.94–2.14)	1.55 (1.04–2.33)
Diabetes	32.20%	33.33%	0.97 (0.6–1.48)	0.96 (0.6–1.47)
Abnormal echocardiogram	18.64.%	16.66%	1.09 (0.6–1.74)	1.05 (0.58–1.7)
Pulmonary disease	20.33%	16.66%	1.17 (0.67–1.83)	1.14 (0.65–1.8)
Kidney dysfunction	30.50%	24.07%	1.23 (0.77–1.86)	1.17 (0.71–1.86)
Ascites and Pleural effusion	42.50%	31.7%	1.39 (0.95–2.01)	1.15 (0.76–1.73)
Sodium (mEq/L)^a^	136 (132–139)	138 (134–140)	0.97 (0.94–1.01)	0.99 (0.95–1.02)
Glomerular filtration rate (mL/min)^a^	86 (60–106)	89 (68.8–121)	1 (0.99–1)	1 (0.99–1)
Creatinine (mg/dl)^a^	0.90 (0.77–1.23)	0.88 (0.74–1.12)	0.99 (0.75–1.19)	0.9 (0.67–1.11)
MELD score^a^	18.5 (12–25)	16.0 (10–21)	1.03 (1.01–1.06)	-
Child–Pugh score				
A	22.07%	35.67%	Reference	Reference
B	25.97%	30.65%	1.46 (0.85–2.58)	1.36 (0.77–2.47)
C	46.75%	33.66%	1.81 (1.12–3.08)	1.56 (0.83–2.99)
Patient baseline data				
Hemoglobin (g/L)^a^	92.50 (82.8–111)	115.00 (94–130)	0.98 (0.97–0.99)	0.98 (0.97–0.99)
Platelet count (10^3^/mm^3^)^a^	75.0 (51.0–113)	83.0 (54–124)	1 (0.99–1)	1 (1–1)
Fibrinogen (g/L)^a^	1.98 (1.40–2.64)	2.52 (1.60–3.38)	0.77 (0.64–0.91)	0.83 (0.67–1.01)
ExTem^a^				
Coagulation time (s)	67.5 (60–77.5)	65 (60–74)	1 (1–1.01)	1 (0.9–1.01)
MCF (mm)	50.5 (44.0–56.0)	55.0 (48.0–63.0)	0.99 (0.97–1.01)	1 (0.98–1.01)
A10 FibTem MCF (mm)	11 (7.0–15.0)	11 (8.0–15.0)	0.99 (0.95–1.02)	1 (0.97–1.03)
Donor data				
Brain death	68.0%	69.76%	Reference	Reference
Cardiac death	32.0%	30.24%	1.08 (0.71–1.58)	1.13 (0.77–1.67)
Intraoperative data				
Temporary portocaval shunt	45.00%	40.97%	1.13 (0.77–1.63)	1.02 (0.7–1,48)
Cold ischemia time (min)^a^	377 (312–444)	370 (212–438)	1 (1–1)	1 (1–1)
Warm ischemia time (min)^a^	37.00 (30.00–54.50)	40 (30.00–52.00)	1 (1–1)	1 (1–1)
Reperfusion syndrome	57.50%	30.24%	2.27 (1.57–3.35)	2.11 (1.43–31)
Transfusion during LT				
RBC (units)^a^	2 (0–5)	0 (0–2)	1.04 (1.04–1.05)	1.04 (1.04–1005)
RBC risk cut point > 2.5 units	48.75%	23.90%	2.13 (1.48–3.06)	1.96 (1.29–2.96)
Patient RBC required, by units				
0	30.00%	57.07%	0.34 (0.2–0.64)	0.37 (0.2–0.72)
1–6	57.50%	38.04%)	0.74 (0.48–1.32	0.75 (0.48–1.34)
>6	12.50%	4.87%	1.89 (1.07–2.87)	1.64 (1.02–2.63)
Fibrinogen concentrate (g)^a^	4.00 (0.00–9.00)	0.00 (0.00–4.00)	1.07 (1.06–1.09)	1.07 (1.05–1.09)
Tranexamic acid, Yes	48.75%	40.0%	1.29 (0.89–1.87)	1.23 (0.85–1.77)
Crystalloids and Albumin (mL)^a^	2,900 (1,970–3,891)	2,000 (1,876–3,500)	1 (1–1)	1 (1–1)

aRR, RR adjusted by MELD score; ExTem, extrinsic thromboelastometry for fibrin tissue factor activation; FibTem, thromboelastometry for fibrin tissue factor activation and platelet inhibition; MCF, maxim clot firmness; MELD, model for end-stage liver disease; RBC, red blood cells; RR, relative risk.

a Data are percentages of patients, or median (interquartile range).

Sex, age, and preoperative co-morbidities were not associated with CDC 3–5 status. A MELD score was associated with risk for CDC 3–5, and a score cut point of >23 showed an even stronger association (aRR 1.92; 95% CI, 1.28–2.8). In contrast, a high plasma concentration of hemoglobin and fibrinogen levels (RR 0.77; 95% CI, 0.64–0.91) were not associated with risk for CDC 3–5, but after adjustments for MELD score, only a baseline hemoglobin concentration (aRR, 0.98; 95% CI, 0.97–0.99) continued to be associated with this outcome.

Zero RBC transfusion was not associated with risk for CDC 3–5 status (aRR 0.37; 95% CI, 0.28–0.72). The RBC transfusion cut point of >2.5 units during LT was found to be strongly associated with CDC 3–5 grade (aRR, 1.96; 95% CI, 1.29–2.96) (Table 3). We found no associations between CDC 3–5 status and use of a temporary PCS, CIT, WIT, surgical time, or intraoperative fluid administration. On the other hand, PRS (aRR, 2.11; 95% CI, 1.43–3.10) and fibrinogen administration (aRR, 1.07; 95% CI, 1.05–1.09) were associated with CDC 3–5 status.

## Discussion

The incidences of CDC 3–5 status and AKI were similar to findings reported for other series (17, 18).

A higher MELD score was associated with CDC 3–5 status, whereas baseline creatinine, glomerular filtration rate, and plasma sodium level were not associated with risk for the outcome. Higher baseline hemoglobin concentration protected against both CDC 3–5 status.

After MELD adjustment, our findings confirmed the relevance of preoperative anemia, consistent with two retrospective series in which anemia was linked to major complications and 90-day and 1-year mortality (19–21). Although mortality was associated with hemoglobin concentration in a series of cirrhotic patients, MELD score was a stronger predictor of death or need for LT in that study (22). In our cohort, the median for the highest quartile of patients with CDC 3–5 status was 111 g/L; in contrast the median value in CDC 0–2 patients was 115 g/L. This value for the patients with fewer complications suggests a target for a testable preoperative optimization strategy, even though optimization is clinically challenging in patients with liver disease (23).

CDC 3–5 patients had altered hemostatic and coagulation and thromboelastometry profiles. High values of PT/INR were associated with CDC 3–5 grading. However, because PT/INR was collinear with the MELD score, it was excluded from analysis, even though both can be considered to confer patient risk for CDC 3–5 status. Baseline plasma concentration of fibrinogen was also associated with CDC 3–5 status, but none of the variables derived from ExTem or FibTem were confirmed.

Duration of surgery, surgical technique, ischemia times, and donor characteristics were not associated with CDC 3–5 status. Because a temporary PCS was performed in nearly 42% of the cohort overall (without differences between the two CDC strata), it is uncertain whether this technique could have influenced the outcome. A meta-analysis of studies of LT using grafts from cadaveric donors reported that using a temporary PCS reduced blood component usage but had no effect on postoperative outcomes (24). However, a study reported that this procedure led to better intraoperative hemodynamic parameters and a decrease in the incidence of reperfusion syndrome (25). The duration of cold ischemia and the absence of a PCS were independent predictors of PRS in that cohort; nevertheless, the percentage of PRS in patients who underwent PCS was higher than expected, at 65% (26). A very recent randomized trial in living donor LT recipients claimed clear hemodynamic advantages for patients with a PCS (27); however, in the control group, inferior vena cava injury was present in a higher-than-expected percentage (26.67% vs. 3.3% in the intervention group), and consequently, intraoperative blood loss, hepatectomy time, total duration of surgery, and blood component requirements were significantly different in the PCS and control groups, calling into question the generalizability of the conclusions to other populations.

In patients with CDC 3–5 status, more patients in this stratum of the cohort also required administration of tranexamic acid at reperfusion of the graft. The occurrence of PRS, which developed in 54.26% of patients in the CDC 3–5 stratum. PRS is caused by liver graft and recipient risk factors, which can coexist. Avoiding hypervolemia and maintaining an optimal plasma calcium level before reperfusion of the graft are keys to mitigating these effects. In addition, the degree of steatosis of the graft is a determining factor in the incidence of PRS and subsequent complications (28). One group's retrospective study found that preconditioning grafts with hypothermic oxygen perfusion machines was associated with a lower incidence of PRS (29).

The relatively low number of LTs analyzed represents a partial limitation of this study. Nevertheless, the size of our series of patients waiting for liver grafts reflects numbers that are typical for most European registries (30, 31). We did not calculate the Comprehensive Complication Index (CCI) which allows a continuous stratification of the outcome and compared with the CDC; the CCI allows a grading system for surgical complications, its predictive value is superior for hospital stay and surgical strategies such as the use of temporary PCS (32). However, the objective of our study was to assess complications and their relationship with baseline patient's characteristic and intraoperative incidents. For this reason, we evaluated the simplicity of data collection of the CDC concomitantly with the course of patients in the critical care unit, seeking factors of association.

Certain variables (portal thrombosis, previous sodium values, PT/INR, and ascites) can be related to the MELD score; however, only PT/INR needed to be excluded because of collinearity. We found no other collinearities and are therefore confident that baseline preoperative hemoglobin and perioperative hemodynamic instability and blood component requirements are the most important factors affecting outcome in this prospective cohort. A third limitation is related to the timing of events. The 20 patients in whom complications occurred after discharge from the ICU and who were readmitted to the ICU were not included in the logistic regression analysis, thus simplifying the model and avoiding the need for right-censoring or time-to-event analysis. However, we note that we found similar results when we did a *post-hoc* analysis of the full group of 97 patients with CDC 3–5 status.

Strengths of the study are the participation of three high-volume LT hospitals, prospective data collection, high adherence to protocols, and the monitoring of data quality by an independent committee.

We hypothesize that despite the challenges of correcting preoperative anemia in liver disease, intravenous iron infusion in wait-listed patients may be feasible. Whether this strategy could possibly reduce blood transfusion remains to be tested in a randomized controlled trial.

We conclude that PRS at reperfusion of the liver graft and the volume of RBCs transfused are the main modifiable factors that influence major complications reflected by CDC 3–5 status after LT.

## Data Availability

The raw data supporting the conclusions of this article will be made available by the authors, without undue reservation.
